# Influence of a Commercial Biological Fungicide containing *Trichoderma harzianum* Rifai T-22 on Dissipation Kinetics and Degradation of Five Herbicides in Two Types of Soil

**DOI:** 10.3390/molecules25061391

**Published:** 2020-03-18

**Authors:** Ewa Szpyrka, Magdalena Podbielska, Aneta Zwolak, Bartosz Piechowicz, Grzegorz Siebielec, Magdalena Słowik-Borowiec

**Affiliations:** 1 University of Rzeszow, Institute of Biology and Biotechnology, 1 Pigonia St., 35-310 Rzeszów, Poland; magdapodbiel@gmail.com (M.P.); azwolak@ur.edu.pl (A.Z.); bpiechow@univ.rzeszow.pl (B.P.); m.slowik_borowiec@interia.pl (M.S.-B.); 2 The Institute of Soil Science and Plant Cultivation, Department of Soil Erosion and Land Conservation, 8 Czartoryskich St., 24-100 Puławy, Poland; gs@iung.pulawy.pl

**Keywords:** herbicide, biodegradation, *Trichoderma harzianum*, half-life

## Abstract

Biological crop protection is recommended to be applied alternately or together with chemical one, to protect human health from the excessive use of toxic pesticides. Presence of microorganisms can influence the concentration of chemical pollutants in soil. The aim of this study is to estimate the influence of a commercial biological fungicide containing *Trichoderma harzianum* Rifai T-22 on dissipation kinetics and degradation of five herbicides belonging to different chemical classes: clomazone, fluazifop-P-butyl, metribuzin, pendimethalin, and propyzamide, in two types of soil. Results of the study revealed that *T. harzianum* T-22 influences pesticide degradation and dissipation kinetics of the non-persistent herbicides: clomazone, fluazifop-P-butyl, and metribuzin. In soil with a higher content of nitrogen, phosphorus, and organic matter, degradation increased by up to 24.2%, 24.8%, and 23.5% for clomazone, fluazifop-P-butyl, and metribuzin, respectively. In soil with lower organic content, degradation was on a low level, of 16.1%, 17.7%, and 16.3% for clomazone, fluazifop-P-butyl, and metribuzin, respectively. In our study, the addition of the biological preparation shortened herbicide dissipation half-lives, from 0.3 days (2.9%) for fluazifop-P-butyl, to 18.4 days (25.1%) for clomazone. During the degradation study, no significant differences were noticed for pendimethalin, belonging to persistent substances. Biological protection of crops can modify pesticide concentrations and dissipation rates. On one hand, this may result in the reduced effectiveness of herbicide treatments, while on the other, it can become a tool for achieving cleaner environment.

## 1. Introduction

Pesticides are substances used worldwide, especially in the plant production, to combat weeds, pests, and diseases, and thence to increase the yield. After fulfilling their task, a majority of pesticides remain in plants, soil, and other components of the environment, and can be uptaken by a consumer, mainly with food or water [1,2].

Because of the concerns related to the environment and human health, it is recommended to use biopesticides in plant protection. Nevertheless, today biopesticides account for about 5% of the total crop protection market. The number of biopesticides registered in the European Union (EU) is lower than that in other countries, like China, India, or the United States, and this results from a complex registration processes [3]. In 2015, 36 microorganisms were approved for the use in pest control in the EU [4]. This small share of biological methods also results from a fact that biopesticides are usually less effective than chemical pesticides, and their application is more complicated [5].

Chemical pesticides are often persistent pollutants, accumulating in the environment. They could be degraded by physical, chemical, or photochemical processes, and also by microorganism [6]. Among biological organisms, bacteria such as *Pseudomonas*, *Bacillus*, *Alcaligenes Flavobacterium*, *Escherichia coli*, *Clostridium*, and *Thiobacillus*, are those with the highest capacity to degrade pesticides, even so persistent as dichlorodiphenyltrichloroethane (DDT). Other types of microorganism capable to decompose these xenobiotics are: *Actinomycetes* (*Micromonospora*, *Actinomyces*, *Nocardia*, *Streptomyces*), fungi (*Rhizopus*, *Cladosporium*, *Penicillium*, *Aspergillus*, *Fusarium*, *Mucor*, *Trichoderma* spp., *Mortierella* sp.), algae (small green algae, *Chlamydomonas*, a genus of diatoms), and yeast [7,8].

Apart from pesticides, microfungi may transform other persistent xenobiotics: aromatic and aliphatic hydrocarbons, including polycyclic aromatic hydrocarbons, nitro aromatics, plasticizers, dibenzofurans, and biphenyls. Filamentous fungi may use two types of enzymatic systems for degradation of these substances: intracellular (cytochromes P450) and exocellular (peroxidases and laccases) [6].

*Trichoderma* spp. are microscopic soilborne filamentous fungi belonging to the division *Ascomycota*, widespread in the world and described for the first time in 1794. Fungi successfully colonize their habitats and efficiently combat their competitors [9]. *Trichoderma* spp. can colonize aboveground and belowground plant organs, and are present between living cells. They are used as biopesticides because of their ability to destroy other fungi and certain nematodes, induce resistance to plant pathogens, impart abiotic stress tolerance, improve plant growth and vigor, solubilize plant nutrients, and bioremediate heavy metals and environmental pollutants. Furthermore, they can produce secondary metabolites and enzymes: chitinases, β-glucanases, cellulases, and protease, which found application in industry. Nowadays, 60% of registered bio-fungicides are based on *Trichoderma* [10,11].

*Trichoderma* spp. can degrade persistent organochlorine pesticides: endrin, aldrin, and DDT [12], endosulfan [13,14], organophosphate: chlorpiryfos [15,16], fenitrothion, fenitrooxon [17], paration methyl [14], and benzimidazole, like carbendazim [18].

Currently, eight strains of *Trichoderma* are used in plant protection products in the EU [19]. One of them is *T. harzianum* Rifai strain, which is recommended against soil and foliar pathogens, including *Pythium*, *Rhizoctonia*, *Fusarium*, and *Botrytis*. Its target hosts are a wide range of crops including: vegetables (tomato, cucumber, and lettuce), ornamentals, soybean, rice, sugarcane, and maize. *T. harzianum* Rifai T-22 develops well in various environmental conditions, in a wide temperature range (8–34 °C), pH of 4–8.5, on various types of substrates and roots of many plant species [20,21].

Nowadays, increasingly more emphasis is being placed on the use of biological agents in the plant cultivation, either combined with or instead of toxic chemical pesticides. In the integrated crop protection, biological control is recommended with or instead of chemical control. Such situation raises questions if applied microorganisms can change the concentration of chemical pesticides. This paper presents the influence of a new commercial biological fungicide containing *T. harzianum* Rifai T-22 on dissipation kinetics and degradation of five herbicides currently approved for the use in the EU, which are highly toxic to humans: clomazone, fluazifop-P-butyl, metribuzin, pendimethalin, and propyzamide (Table 1). These biological and chemical preparations are registered in Poland for protection of certain vegetables: lettuce, tomatoes, and cucumbers [22]. In literature, no information is available on the influence of that fungal strain on the fate of those chemical substances in soil.

## 2. Results

Dissipation parameters (kinetic equations with correlation coefficients and half-lives, t_½_) of five tested herbicides in two types of soil without and with *T. harzianum* Rifai T-22 added are presented in Table 2. Experiment 1 was conducted in soil 1 and experiment 2 was conducted in soil 2.

In experiment 1, the initial values found for the herbicides were higher, and this can be associated with soil 1 properties and higher sorption of pesticides. Soil 1 has higher organic content (humus content of 69.9 ± 0.5%, vs. 55.6 ± 0.4% in soil 2) and smaller particles (0–5 mm, vs. 0–30 mm in soil 2) (Table 3). Pesticide dissipation was described by exponential equations of (pseudo) first-order kinetics, in which the initial concentration does not affect the half-life [25,26].

### 2.1. Clomazone

Clomazone belongs to the isoxazolidinones, from Group F3 (carotenoid biosynthesis inhibitors) according to the Herbicide Resistance Action Committee (HRAC). It is a non-persistent herbicide, moderately mobile in soil (Table 1) [24].

In experiment 1, clomazone dissipated according to equations: y = 25.0012e^−0.0078x^ (0.8889) and y = 23.6252e^−0.0084x^ (0.9725), and half-lives of its active substances were 88.8 and 82.5 days in soil without and with *T. harzianum* Rifai T-22 added, respectively (Table 2). In the soil with the fungi added, levels of clomazone residues were lower than in control samples, ranging from 0.2% on day 29 to 24.2% on day 1. In experiment 1, the statistically significant value *p* < 0.01 (**) was found only on day 64 (Figure S1a, Supplementary material).

In experiment 2, clomazone dissipated according to equations: y = 18.4837e^−0.0095x^ (0.9374) and y = 18.7542e^−0.0127x^ (0.9546), and half-lives of its active substances were 72.9 and 54.6 days in soil without and with *T. harzianum* Rifai T-22 added, respectively (Table 2). The highest degradation of clomazone residues was noted on day 64 of the experiment, reaching a level of 16.1%. In experiment 2, the statistically significant values *p* < 0.05 (*) were seen on day 29 and day 64 (Figure S1b, Supplementary material).

Observed differences in clomazone concentrations in control samples versus samples containing *Trichoderma* were higher in experiment 1 (a maximum of 24.2%) than in experiment 2 (a maximum of 16.1%), in the soil containing organic matter, which has a positive influence on the degradation rate [27]. A faster clomazone dissipation rate (lower t_½_ values), however, was observed in soil 2 (Table 2), and this could be associated with larger particles (0–30 mm fraction), lower organic matter content, and lower pesticide sorption, improving its bioavailability [27,28].

For clomazone a typical laboratory half-life at 20 °C is 22.6 days (6.3–145.7 days) and its field half-life is 27.3 days (9.3–195 days) [24]. This wide spread in half-life values suggests that clomazone dissipation may be influenced by many factors, including UV irradiation [29], and the presence of oxygen [30] or ammonia-oxidizing bacteria [31]. In our study, the addition of the biological preparation shortened the dissipation half-lives by 6.3 days (7.1%) (experiment 1) and 18.4 days (25.1%) (experiment 2). Reis et al. [32] studied herbicide impact on strains of *Trichoderma* spp. They found that clomazone can reduce mycelia growth by up to 30% for some isolates, but for others it had stimulatory effect on fungi sporulation.

### 2.2. Fluazifop-P-butyl

Fluazifop-P-butyl belongs to aryloxyphenoxypropionates, from HRAC Group A1 (acetyl CoA carboxylase (ACCase) inhibitors). It is non-persistent and slightly mobile in soil (Table 1) [24].

In experiment 1, fluazifop-P-butyl dissipated according to equations: y = 5.0777e^−0.0660x^ (0.7800) and y = 4.4082e^−0.0680x^ (0.7743), and half-lives of its active substances were 10.5 and 10.2 days in soil without and with *T. harzianum* Rifai T-22 added, respectively (Table 2). In soil with fungi added, fluazifop-P-butyl residues were at a lower level compared to control samples, in the range of 1.3–24.8%. In experiment 1, the statistically significant values *p* < 0.05 (*) were found on days 8, 15, 29, and 43 (Figure S2a, Supplementary material).

In experiment 2, fluazifop-P-butyl dissipated according to equations: y = 2.8792e^−0.0573x^ (0.7594) and y = 3.7225e^−0.0637x^ (0.8226), and half-lives of its active substances were 12.1 and 10.9 days, in soil without and with *T. harzianum* Rifai T-22 added, respectively (Table 2). During the first month, higher levels of this herbicide were found in soil with *Trichoderma*, and this can be associated with soil acidification by fungi and an increase in fluazifop-P-butyl stability [33]. During the second month of the experiment, degradation of fluazifop-P-butyl residues was determined to be at a level of up to 17.7%. In experiment 2, the statistically significant value *p* < 0.01 (**) was observed on day 8, and values with *p* < 0.05 (**) were found on day 15 and 43 (Figure S2b, Supplementary material).

Similarly as in the case of clomazone, observed differences in the fluazifop-P-butyl concentrations in control samples versus samples containing *Trichoderma* were greater in experiment 1 (a maximum of 24.8%) than in experiment 2 (a maximum of 17.7%), in the soil containing organic matter, which has a positive influence on degradation rate [27].

A typical laboratory half-life of fluazifop-P-butyl at 20 °C is 1.0 days (0.3–3.3 days) and its field half-life is 8.2 days (2.1–38.0 days) [24]. In our study, t_½_ values were very similar in all experiments, but the addition of the biological preparation shortened the dissipation half-lives by 0.3 days (2.9%) (experiment 1) and 1.2 days (9.9%) (experiment 2) (Table 2). In the fluazifop-P-butyl breakdown, pH and the microbial degradation seemed to predominate [24,27]. Fluazifop-P-butyl does not inhibit *T. harzianum* growth [34].

### 2.3. Metribuzin

Metribuzin belongs to triazinones, from HRAC Group C1 (photosystem II inhibitor). It is non-persistent and mobile in soil (Table 1) [24].

In experiment 1, metribuzin dissipated according to equations: y = 681.8803e^−0.0139x^ (0.9797) and y = 715.2762e^−0.0170x^ (0.9436), and half-lives of its active substances were 49.9 and 40.8 days in soil without and with *T. harzianum* Rifai T-22 added, respectively (Table 2). On the first sampling day, the metribuzin concentration was higher by 23.7% in soil with fungi versus the control samples, and then it was reduced by up to 23.5% after 3 weeks. In experiment 1, no statistically significant differences were found between samples with and without *Trichoderma* (Figure S3a, Supplementary material).

In experiment 2, metribuzin dissipated according to equations: y = 529.6056e^−0.0190x^ (0.9045) and y = 540.8551e^−0.0222x^ (0.9097), and half-lives of its active substances were 36.5 and 31.2 days in soil without and with *T. harzianum* Rifai T-22 added, respectively (Table 2). On the first sampling day, similarly as in experiment 1, the metribuzin concentration was higher by 10.9% in soil with fungi versus the control samples, and then it was reduced by up to 16.3% after 6 weeks. In experiment 2, the statistically significant value *p* < 0.05 (*) was observed only on day 1 (Figure S3b, Supplementary material).

Similarly as in the case of clomazone and fluazifop-P-butyl, observed differences in metribuzin concentrations in control samples versus samples containing *Trichoderma* were greater in experiment 1 (maximum 23.5%) than in experiment 2 (maximum 16.3%), in the soil containing organic matter, which has a positive influence on degradation [27]. Similarly as in the case of clomazone, faster metribuzin dissipation rate (lower t_½_ values) was observed in soil 2, and this could be associated with larger particles (fraction 0–30 mm), lower organic matter content, and lower pesticide sorption, improving its bioavailability [27,28]. In our study, the addition of the biological preparation shortened dissipation half-lives by 9.1 days (18.2%) (experiment 1) and 5.3 days (14.5%) (experiment 2).

According to PPDB database, a typical laboratory half-life of metribuzin at 20 °C is 7.1 days (4.73–12.5 days) and its field half-life is 19 days [24], while according to the EFSA document [26], its laboratory half-life is in the range of 5.3–48.8 days. The temperature, bacteria present, and a pH value also have impact on metribuzin dissipation [35]. Metribuzin does not suppress the mycelial growth of *T. harzianum* [36], and fungi are able to biodegrade this herbicide [37].

### 2.4. Pendimethalin

Pendimethalin is a dinitroaniline herbicide belonging to HRAC Group K1 (mitosis inhibitor). It is a persistent and non-mobile substance (Table 1) [24].

In experiment 1, pendimethalin dissipated according to equations: y = 3534.1050e^−0.0154x^ (0.9010) and y = 3040.6923e^−0.0121x^ (0.9019), and half-lives of its active substances were 45.0 and 57.3 days in soil without and with *T. harzianum* Rifai T-22 added, respectively (Table 2). On the first sampling day, the pendimethalin concentration in soil with fungi was lower by 21.1% versus the control samples. On subsequent sampling days, similar pendimethalin concentrations were found in control samples and samples with *Trichoderma*. In experiment 1, no statistically significant differences were observed between samples with and without fungi (Figure S4a, Supplementary material).

In experiment 2, pendimethalin dissipated according to equations: y = 2675.0044e^−0.0148x^ (0.9272) and y = 2497.1433e^−0.0147x^ (0.8960), and half-lives of its active substances were 46.8 and 47.1 days in soil without and with *T. harzianum* Rifai T-22 added, respectively (Table 2). On the first sampling day and on day 29 after treatment, concentrations of pendimethalin were 2.3% and 3.5% higher in soil with fungi versus the control samples, respectively, and on all other sampling days its concentrations were lower, by up to 14.7% on day 29. In experiment 2, no statistically significant differences were observed between samples with and without fungi (Figure S4b, Supplementary material).

A typical laboratory half-life of pendimethalin at 20 °C is 182.3 days (97–270 days), and its field half-life is 100.6 days (39.8–187 days) [24]. A typical field half-life of 44 days was reported, but noted to range from 90 to 480 days depending on the soil temperature, while moisture and biodegradation are not important environmental factors influencing future processes associated with pendimethalin. Additionally, biodegradation may be attenuated by its strong adsorption to soil particles, causing its lower bioavailability [23]. Kočárek et al. [38] studied the effect of a complex of microbial strains containing lactic acid bacteria, yeast, fungi, gram-positive actinomycetes, and photosynthetic bacteria. They did not found any significant influence on the pendimethalin half-life. Ries et al. [32] observed inhibitory effects of pendimethalin on the radial mycelial growth (even up to 66%) of some isolates of *Trichoderma* which could be associated with herbicide properties—a direct interference with cell division, preventing the formation of microtubules responsible for chromosome segregation during mitosis. In our study, the addition of biological preparation extended the half-life dissipation by 12.3 days (27.3%) (experiment 1) and 0.3 day (0.6%) (experiment 2) (Table 2).

### 2.5. Propyzamide

Propyzamide belongs to benzamides and HRAC Group K1 (mitosis inhibitor). It is moderately persistent and slightly mobile in soil (Table 1) [24].

In experiment 1, propyzamide dissipated according to equations: y = 1398.8502e^−0.0204x^ (0.8942) y = 1426.6253e^−0.0181x^ (0.8746), and half-lives of its active substances were 34.0 and 38.3 days in soil without and with *T. harzianum* Rifai T-22 added, respectively (Table 2). Differences in propyzamide concentrations observed between the samples with fungi and the controls were in a range of 6.6–31.1% on relevant sampling dates. In experiment 1, the statistically significant value *p* < 0.01 (**) was found only on day 64 (Figure S5a, Supplementary material).

In experiment 2, propyzamide dissipated according to equations: y = 763.2167e^−0.0128x^ (0.9664) and y = 783.1032e^−0.0165x^ (0.9625), and half-lives of its active substances were 54.1 and 42.0 days in soil without and with *T. harzianum* Rifai T-22 added, respectively (Table 2). On first two sampling dates, propyzamide levels were 0.3% and 7.4% higher in soil with fungi versus the control samples, respectively, and on all others sampling days its concentrations were lower, by up to 23.9% on day 43. In experiment 2, the statistically significant value *p* < 0.05 (*) was observed only on day 43 (Figure S5b, Supplementary material).

Similarly as in the case of clomazone, fluazifop-P-butyl and metribuzin, observed differences in propyzamide concentrations in control samples versus samples containing *Trichoderma* added were greater found in experiment 1 (maximum 31.1% on day 29) than in experiment 2 (maximum 23.9%), in the soil containing organic matter which has a positive influence on degradation [27]. In contrary to clomazone, fluazifop-P-butyl and metribuzin, faster propyzamide dissipation rate (lower t_½_ values of) was observed in soil 1 than in soil 2 (Table 2).

A typical laboratory half-life of propyzamide at 20 °C is 50.5 days (13.9–271.3 days), and its field half-life is 233 days [24]. The persistence of propyzamide varies, depending on a soil type, climatic conditions, and decomposition; and it decomposes by photodecomposition and volatilization from soil surfaces under very hot and dry conditions [23]. The addition of mineral fertilizers to soil inhibited propyzamide degradation by decreasing its resistivity [39]. In our study, after the biological preparation was added to soil, the substance half-life was extended by 4.3 days (12.6%) (experiment 1), and reduced by 12.1 days (22.4%) (experiment 2) (Table 2). Such inconclusive data imply a need for further research into the behavior of these pesticides in soil.

During the GC-MS analysis, only active substances of all herbicides were found in soil samples, and no metabolites were found by the GC-MS technique applied in the full scan mode. It was probably due to the fact that during pesticide degradation in a highly humid (57–64%) soil, more polar metabolites can form, which cannot be determined by the applied GC-MS technique with a nonpolar HP-5 MS column.

## 3. Discussion

Many factors can influence pesticide degradation in soil: type of soil, its content of mineral and organic matter, pH, moisture, temperature, presence of microorganisms, and chemical structure of active substances. It is reflected in half-lives of herbicides, which can vary significantly between studies. For three out of five tested pesticide: clomazone, fluazifop-P-butyl, and metribuzin, which are non-persistent, during the study on their degradation significant differences were noted between samples with the biological preparation containing *T. harzianum* T-22 and the control. In soil 1, in which humus content was higher by 14% versus soil 2, the degradation was higher by up to 24.2%, 24.8%, and 23.5% for clomazone, fluazifop-P-butyl, and metribuzin, respectively. In soil 2, a lower level of degradation occurred, of 16.1%, 17.7%, and 16.3% for clomazone, fluazifop-P-butyl, and metribuzin, respectively. In our study, the addition of the biological preparation shortened herbicide dissipation half-lives, from 0.3 days (2.9%) for fluazifop-P-butyl (experiment 1) to 18.4 days (25.1%) for clomazone (experiment 2). In experiment 1, higher values were found for all herbicides, and this can be associated with greater active substance adsorption by organic matter and smaller size of the soil fraction.

Many studies concern pesticide degradation by *Trichoderma* spp., but the majority of them are conducted in culture media in laboratory conditions propitious for fungal growth. *T. viride* can degrade several organophosphorus (fenitrothion, fenitrooxon, parathion methyl), carbamate, and chlorinated hydrocarbon insecticides: DDT, endrin, aldrin [12,14,17,40]. In studies conducted by Katayama and Matsumura [13], *T. harzianum*, was found to degrade DDT, dieldrin, endosulfan, pentachloronitrobenzene, and pentachlorophenol, but not hexachlorocyclohexane. The authors concluded that the oxidative system is the main enzyme system responsible for pesticide degradation. Jayaraman et al. [15] stated that *T. viride* and *T. harzianum* and its consortium were able to grow in fungal culture medium in the presence of chlorpyrifos, and they demonstrated an increase in the level of biomass and protein production. Harish et al. [16] found that *T. harzianum* and *Rhizopus nodosus* isolated from the contaminated soil can degrade up to 70–80% of chlorpyrifos and ethion in 21 days. Sharma et al. [18] demonstrated the *Trichoderma* spp. degradation capacity versus carbendazim, a benzimidazole-based fungicide. After 5 days, biodegradation of this active substance reached 85% for *T. harzianum*, 47% for *T. viride*, and 21% for *T. atroviride*.

In our study, no significant differences were noted between samples with the biological preparation containing *T. harzianum* T-22 and the control during the study on degradation of a persistent herbicide like pendimethalin. For pendimethalin, the biological preparation extended the dissipation half-life by 12.3 days (27.3%) (experiment 1) and 0.3 days (0.6%). In the case of propyzamide, which is moderately persistent, its half-life was extended by 4.3 days (12.6%) (experiment 2) and shortened by 12.1 days (22.4%) (experiment 2) (Table 2). Such inconclusive data imply a need for further research into the behavior of these pesticides in soil.

## 4. Materials and Methods

The characteristics of herbicides tested: its octanol-water partition coefficient, chemical structure and group, HRAC group, mode of action, soil degradation, mobility, laboratory and field half-lives, and possible metabolites in soil are presented in Table 1.

### 4.1. Reagents

The reagents used for analyses of herbicides in soil included acetonitrile 400g/L and acetone of analytical grade (CPAchem, Bulgaria-Frence), petroleum ether for GC (Chempur, Polska), analytical standards of herbicides (Sigma-Aldrich, USA), QuEChERS Extraction Kits (4 g magnesium sulfate, MgSO_4_; 1 g sodium chloride, NaCl; 1 g sodium citrate, Na_3_C_6_H_5_O_7_; 0.5 g disodium citrate sesquihydrate, C_12_H_18_Na_4_O_17_), and QuEChERS Dispersive Kits (150 mg of primary and secondary amines PSA and 900 mg, MgSO_4_) (Agilent Technologies, Palo Alto, CA, USA).

### 4.2. Soil Samples Preparation

Two types of soil suitable for vegetable cultivation were tested.

Soil 1 was a horticultural soil recommended for vegetable production. It was very acidic (pH 4.6), with a high content of organic carbon (OC) and total nitrogen (N). Soil 2 was a universal peat substrate mixed with perlite, acidic, but with the pH value slightly higher than in Soil 1. It was highly organic soil with the OC and N content only slightly lower than in Soil 1. Soil 2 had a moderate content of plant available P, while in soil 1 P availability was very high (seven times greater than in Soil 2). Soil parameters: fraction, pH, humus content, total carbon, organic carbon, total nitrogen, assimilable phosphorus, and other elements, were determined in the laboratory of The Institute of Soil Science and Plant Cultivation in (Pulawy, Poland) accredited by The Polish Centre for Accreditation (certificate no AB 339) (Table 3).

The experiments were performed in spring, from 25 March to 30 May, in natural light, when in Poland the average day to night ratio is 15 h to 9 h, respectively. Total of 400 g samples of soil were weighted into transparent propylene containers of 2 L each. The biological fungicide TRIANUM-G (Koppert BV, The Netherlands) [21] containing *T. harzianum* Rifai T-22 was added to one-half of all soil samples at a concentration of 750 g/L m^3^ and mixed, as recommended in the instruction of the commercial product. On the next day, the soil samples were sprayed with 80 mL of commercial herbicides prepared at the highest doses recommended in their labels [22] (Table 4).

Herbicides: Clomazone (Command 480 EC, FMC Chemical, Belgium—1 mL for 1 L of water), metribuzin (Aurelit 70 WG, ADAMA Deutschland GmbH, Germany—2.5 g for 1 L of water), pendimethalin (Stomp Aqua 455 CS, BASF SE, Germany—12.5 mL for 1 L of water), fluazifop-P-butyl (Fusilade Forte 150 EC, Syngenta Polska Sp. z o.o., Poland—17 mL for 1 L of water), and propyzamide (Kerb 50 WP, Dow AgroSciences Polska Sp. z o.o., Poland—5 g for 1 L of water) were applied. Each pesticide was applied separately. The samples were prepared and analyzed in three replicates (three containers for each sample). Propylene containers were closed with a cover one day after chemical spraying and were opened to take samples for GC analysis. The samples for the pesticide analysis were taken after 1, 8, 15, 22, 29, 43, and 64 days. Before sampling, each soil was mixed thoroughly with a laboratory spoon. In each sample, the water content was measured by a weighing method after drying at 105 °C (S–40, Alpina, Poland) [41]. The moisture of soil samples was in the range of 57–64%. The pesticide concentration was calculated for the soil dry mass. All soil samples were stored in stable laboratory conditions. Throughout the experiments, the air temperature was maintained at 22 ± 1 °C.

### 4.3. GC-µECD Analysis of Herbicide Residues

The analysis of herbicides in soil was done using a modified method based on the European Norm and literature on the QuEChERS method for soil analysis, involving sample extraction with acetonitrile and clean-up through a dispersive solid phase extraction (d-SPE) [42,43,44,45]. Total of 5 g of soil were weighted into 50-mL polypropylene centrifuge tube, and 20 mL of acetonitrile 400 g/L (acetonitrile:water 1:1 *v*/*v*) were added, hand shaken for 1 min to hydrate the sample and allowed to stand for 10 min. The tube content was vortexed (BenchMixer^TM^, Benchmark, Edison, NY, USA) for 1 min. Then, a mixture of salts containing 4 g MgSO_4_; 1 g NaCl; 1 g Na_3_C_6_H_5_O_7_, and 0.5 g C_12_H_18_Na_4_O_17_ was added. The tube content was vortexed in a mixer (BenchMixer^TM^, Benchmark, Edison, NY, USA) for 1 min and then centrifuged at 3000 rpm (MPW-350R, MPW MED. INSTRUMENTS, Warszawa, Poland) for 5 min. Total of 5 mL of the organic phase were transferred to a 15-mL polypropylene centrifuge tube that contained sorbent for clean-up, consisting of 150 mg of PSA and 900 mg MgSO_4_. The tube content was vortexed in the mixer (BenchMixer^TM^, Benchmark, Edison, NY, USA) for 1 min and centrifuged at 3000 rpm (MPW-350R, MPW MED. INSTRUMENTS, Warszawa, Poland) for 5 min. Total of 1 mL of purified extract was transferred to a 2-mL chromatographic vial, acetonitrile extract was evaporated to dryness under a gentle stream of pure nitrogen and then dissolved in 1 mL of petroleum ether. Each sample was analyzed in three replicates.

A 7890A gas chromatograph (Agilent Technologies, Palo Alto, CA, USA) equipped with a capillary column (HP-5 MS Ultra Inert/30 m × 0.25 mm I.D. × 0.25-μm) and a micro electron capture detector (GC-µECD) was used to analyze the herbicide residues in soil extracts. The following chromatographic parameters were used: injection of samples in a splitless mode, injected volume—2 μL, carrier gas—helium (5.0 purity, flow of 1.37 mL/min), and the temperatures of 250 °C for the inlet, 300 °C for the detector, and 100–220 °C for the oven. Software ChemStation (Rev. B04.03-SP2, Agilent Technologies, Palo Alto, CA, USA) was used for acquisition and processing of the analysis results.

### 4.4. Confirmatory GC-MS Analysis of Pesticides and Possible Metabolites

A 7890A gas chromatograph (Agilent Technologies, Palo Alto, CA, USA) equipped with a mass detector, model 7000 (GC-MS/MS QqQ), was used to confirm the herbicide residues in soil extracts and search for possible metabolites. The following analysis parameters were used: injection of samples in a splitless mode, injected volume—1 μL, carrier gas—helium (5.0 purity, flow 1 mL/min), ionization mode—electron (−70 eV), and the temperatures of 250 °C for the transfer line, 230 °C for the ion source, 150 °C for the quadrupoles, and 40–260 °C for the oven. Software Mass Hunter, version B.07.06 was used for acquisition and processing of the analysis results. Ions were monitored in the full scan mode. Spectra were compared to spectra obtained for analytical standards of herbicides and to NIST database.

### 4.5. Method Validation

The validation was performed according to the EU guideline SANTE [46]. The parameters of linearity (expressed as a coefficient of determination R^2^), trueness and precision (expressed as an average recovery and a relative standard deviation, RSD), matrix effects, and a limit of quantification (LOQ) were assessed. The materials for validation were soil samples without herbicide residues.

Validation parameters for all active substances in two types of soil met the EU criteria for pesticide analyses described in SANTE documents [46]. Detailed characteristics are given in Table 5. For the herbicide tested, a slight matrix effect was found, ranging from −1 to 11%, except for fluazifop-P-butyl, where it amounted to −42% in soil 1 and −28% in soil 2. Therefore, all analyses were performed with reference to the standard mixture prepared in the “blank” matrix.

### 4.6. Statistical Analysis of Results

The Tukey test (program Statistica version 7) was used to determine statistically significant differences between samples, with and without biological preparation added, for each sampling day. Statistically significant p values are shown in Figures S1–S5, Supplementary material as *p* < 0.05 (*) and *p* < 0.01 (**). No values at a level of *p* < 0.001 (***) were determined.

## 5. Conclusions

*T. harzianum* T-22 influences pesticide degradation and dissipation kinetics for non-persistent herbicides, such as clomazone, fluazifop-P-butyl, and metribuzin. Biological protection of crops can change the concentration of these active substances. On the one hand, this may result in the reduced effectiveness of herbicide treatments, while on the other hand, it can become a tool for achieving the cleaner environment. During the degradation study, no significant differences were noted for pendimethalin which is more persistent. Many other factors can influence the pesticide biodegradation, so further detailed studies should be undertaken in this area.

## Figures and Tables

**Table 1:** Active substances of studied herbicides and their properties [23,24].

Active Substance	Partition Coefficient Octanol-Water log P	Chemical Structure	Chemical Group/HRAC ^1^ Group—Mode of Action	Soil Degradation/Mobility/Laboratory Half-Life/Field Half-Life	Possible Metabolites in Soil
Clomazone	2.58	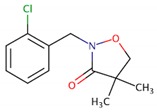	Isoxazolidinone/F3—carotenoid biosynthesis inhibitor	Non-persistent/moderately mobile /6.3–145.7 days/9.3–195 days	(N-((2-chlorobenzyl))-3-hydroxy-2.2-dimethylpropanamide)
Fluazifop-P-butyl	4.5	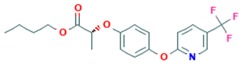	Aryloxyphenoxypropionate/A1—acetyl CoA carboxylase (ACCase) inhibitor	Non-persistent/slightly mobile/0.3–3.3 days/2.1–38.0 days	no
Metribuzin	1.75	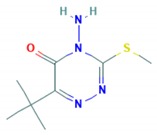	Triazinone/C1—photosystem II inhibitor	Non-persistent/mobile/4.73–12.5 days/19 days	Diketo-metribuzin; desaminodiketo-metribuzin; desamino-metribuzin
Pendimethalin	5.4	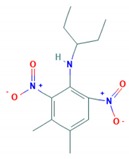	Dinitroaniline/K1—mitosis inhibitor	Persistent/non-mobile/97–270 days/39.8–187 days	2-methyl-3.5-dinitro-4-(pentan-3ylamino)benzoic acid
Propyzamide	3.27	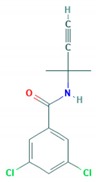	Benzamide/K1—mitosis inhibitor	Moderately persistent/ slightly mobile/13.9–271.3 days/233	2-(3.5-dichlorophenyl)-4.4-dimethyl-5-methylene-oxazoline; N-(1.1-dimethylacetonyl)-3.5-dichlorobenzamide

^1^ HRAC—Herbicide Resistance Action Committee.

**Table 2:** Dissipation parameters of herbicides in soil without and with *T. harzianum* Rifai T-22 added, and differences in their half-lives between soils with and without fungi.

Active Substance	Experiment 1				Experiment 2			
	Equation (R ^1^)	t_½_^2^ (day)	Differences in t_½_^2^ (day)	Differences in t_½_^2^ (%)	Equation (R ^1^)	t_½_^2^ (day)	Differences in t_½_^2^ (day)	Differences in t_½_^2^ (%)
Clomazone	y = 25.0012e^−0.0078x^ (0.8889)	88.8	6.3	7.1	y = 18.4837e^−0.0095x^ (0.9374)	72.9	18.3	25.1
Clomazone + *T. harzianum* Rifai T-22	y = 23.6252e^−0.0084x^ (0.9725)	82.5			y = 18.7542e^−0.0127x^ (0.9546)	54.6		
Fluazifop-P-butyl	y = 5.0777e^−0.0660x^ (0.7800)	10.5	0.3	2.9	y = 2.8792e^−0.0573x^ (0.7594)	12.1	1.2	9.9
Fluazifop-P-butyl + *T. harzianum* Rifai T-22	y = 4.4082e^−0.0680x^ (0.7743)	10.2			y = 3.7225e^−0.0637x^ (0.8226)	10.9		
Metribuzin	y = 681.8803e^−0.0139x^ (0.9797)	49.9	9.1	18.2	y = 529.6056e^−0.0190x^ (0.9045)	36.5	5.3	14.5
Metribuzin + *T. harzianum* Rifai T-22	y = 715.2762e^−0.0170x^ (0.9436)	40.8			y = 540.8551e^−0.0222x^ (0.9097)	31.2		
Pendimethalin	y = 3534.1050e^−0.0154x^ (0.9010)	45.0	12.3	27.3	y = 2675.0044e^−0.0148x^ (0.9272)	46.8	0.3	0.6
Pendimethalin + *T. harzianum* Rifai T-22	y = 3040.6923e^−0.0121x^ (0.9019)	57.3			y = 2497.1433e^−0.0147x^ (0.8960)	47.1		
Propyzamide	y = 1398.8502e^−0.0204x^ (0.8942)	34.0	4.3	12.6	y = 763.2167e^−0.0128x^ (0.9664)	54.1	12.1	22.4
Propyzamide + *T. harzianum* Rifai T-22	y = 1426.6253e^−0.0181x^ (0.8746)	38.3			y = 783.1032e^−0.0165x^ (0.9625)	42.0		

^1^ R—correlation coefficient; ^2^ t_½_—half-life.

**Table 3:** Soils parameters.

Parameter	Soil 1	Soil 2
Type	Horticultural soil recommended for vegetable production	Universal peat substrate mixed with perlite
Fraction	0–5 mm	0–30 mm
pH	4.6 ± 0.1 ^1^	5.3 ± 0.1
Humus content	69.9 ± 0.5%	55.6 ± 0.4%
Total carbon	46.4 ± 0.4%	36.6 ± 0.3%
Organic carbon	40.5 ± 0.4%	32.2 ± 0.4%
Total nitrogen	1.6 ± 0.1%	1.4 ± 0.1%
Assimilable phosphorus	104.5 ± 2.2 mg P_2_O_5_/100g	14.9 ± 0.6 mg P_2_O_5_/100g
Other elements, as mg/kg	Li 0.13 ± 0.01, Be 0.03 ± 0.01, V 1.71 ± 0.30, Cr 1.32 ± 0.30, Mn 25.07 ± 1.80, Co 0.20 ± 0.02, Ni 1.09 ± 0.09, Cu 9.47 ± 1.10, Zn 12.16 ± 2.60, As 1.37 ± 0.10, Se 0.52 ± 0.08, Sr 33.83 ± 4.20, Mo 11.57 ± 1.90, Cd 0.15 ± 0.04, Sb 0.06 ± 0.02, Ba 29.62 ± 2.90, La 0.59 ± 0.12, Ce 1.42 ± 0.30, Eu 0.03 ± 0.01, Gd 0.14 ± 0.02, Tl 0.02 ± 0.01, Pb 5.06 ± 0.80, Bi 0.08 ± 0.01, Na 173.50 ± 8.90, Mg 805.79 ± 19.60, Al 699.63 ± 42.0, K 1080.42 ± 25.0, Ca 14390.90 ± 256.0, Fe 2808.49 ± 86.0	Li 0.48 ± 0.09, Be 0.07 ± 0.02, V 3.26 ± 0.15, Cr 1.95 ± 0.17, Mn 30.74 ± 1.16, Co 0.93 ± 0.06, Ni 4.30 ±0.13, Cu 3.78 ± 0.41, Zn 6.75 ± 0.90, As 2.66 ± 0.11, Se 0.51 ± 0.02, Sr 85.10 ± 2.10, Mo 0.62 ± 0.02, Cd 0.14 ± 0.03, Sb 0.12 ± 0.02, Ba 20.47 ± 1.10, La 1.49 ± 0.08, Ce 2.97 ± 0.50, Eu 0.06 ± 0.02, Gd 0.30 ± 0.08, Tl 0.02 ± 0.01, Pb 4.62 ± 0.70, Bi 0.10 ± 0.03, Na 299.17 ± 9.20, Mg 637.42 ± 14.10, Al 1410.11 ± 65.0, K 431.25 ± 11.20, Ca 22094.49 ± 131.0, Fe 3290.40 ± 47.0

^1^ Standard deviation—SD (three replications of each sample were analyzed).

**Table 4:** Recommended application, based on the labels [22].

Plant Protection Product	Active Substance	Active Substance Content in Product	Recommended Dose	Recommended Water Volume	Application Method
Command 480 EC	clomazone	480 g/L	0.2 L/ha	200–300 L/ha	spray
Aurelit 70 WG	metribuzin	700 g/kg	0.5 kg/ha	200–300 L/ha	
Stomp Aqua 455 CS	pendimethalin	455 g/L	25 mL/100 m^2^	2–4 L/100 m^2^	
Fusilade Forte 150 EC	fluazifop-P-butyl	150 g/L	0.63–1.7 L/ha	100–400 L/ha	
Kerb 50 WP	propyzamide	500 g/kg	1–1.5 kg/ha	300–400 L	
Trianum-G	*T. harzianum Rifai* T-22	10 g/kg	750 g/1 m^3^ of soil	-	mixing with soil

**Table 5:** Validation parameters for herbicide active substances in two kind of soils (GC-µECD).

Active Substance	Linearity (R^2^) ^1^	Average Recovery (±RSD ^2^) (%)		Matrix Effects (±RSD ^2^) (%)	
		Soil 1	Soil 2	Soil 1	Soil 2
Clomazone	0.995	81 (6)	73 (2)	5	−1
Fluazifop-P-butyl	0.990	84 (3)	80 (16)	−42	−28
Metribuzin	0.997	120 (7)	119 (10)	10	11
Pendimethalin	0.999	114 (5)	119 (7)	5	2
Propyzamide	0.999	120 (5)	107 (9)	4	9

^1^ R^2^—coefficient of determination; ^2^ RSD—relative standard deviations (%).

## Supplementary Materials

Supplementary materials can be found online. Figure S1. Plots of clomazone dissipations, A—experiment 1, B—experiment 2. Statistically significant *p* values are shown as *p* < 0.05 (*) and *p* < 0.01 (**). Figure S2. Plots of fluazifop-P-butyl dissipations, A—experiment 1, B—experiment 2. Statistically significant *p* values are shown as *p* < 0.05 (*) and *p* < 0.01 (**). Initial values of fluazifop-P-butyl are provided in plots. Figure S3. Plots of metribuzin dissipations, A—experiment 1, B—experiment 2. Statistically significant *p* value is shown as *p* < 0.05 (*). Figure S4. Plots of pendimethalin dissipations, A—experiment 1, B—experiment 2. Figure S5. Plots of propyzamide dissipations, A—experiment 1, B—experiment 2. Statistically significant *p* values are shown as *p* < 0.05 (*) and *p* < 0.01 (**).
