# Supplementary material for: Influence of a Commercial Biological Fungicide containing Trichoderma harzianum Rifai T-22 on Dissipation Kinetics and Degradation of Five Herbicides in Two Types of Soil
Source: Molecules. 2020 Mar 18;25(6):1391. doi: 10.3390/molecules25061391 (PMC7144550; doi:10.3390/molecules25061391)
Supplement: Supplementary file 1 [file molecules-25-01391-s001.pdf]

Supplementary material

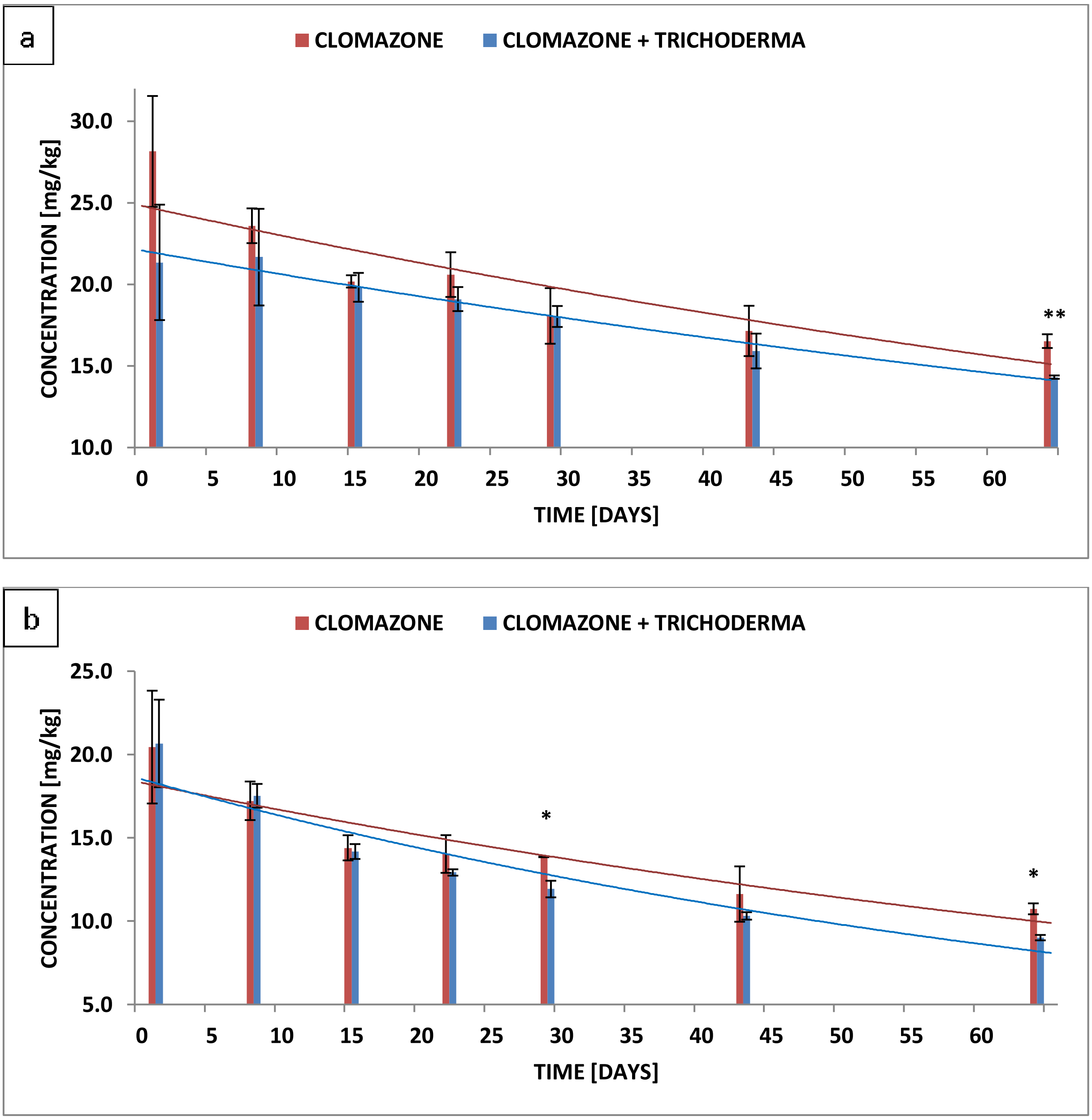

Figure S1. Plots of clomazone dissipations, A - experiment 1, B - experiment 2. Statistically significant p values are shown as $p < 0.05$ (*) and $p < 0.01$ (**).

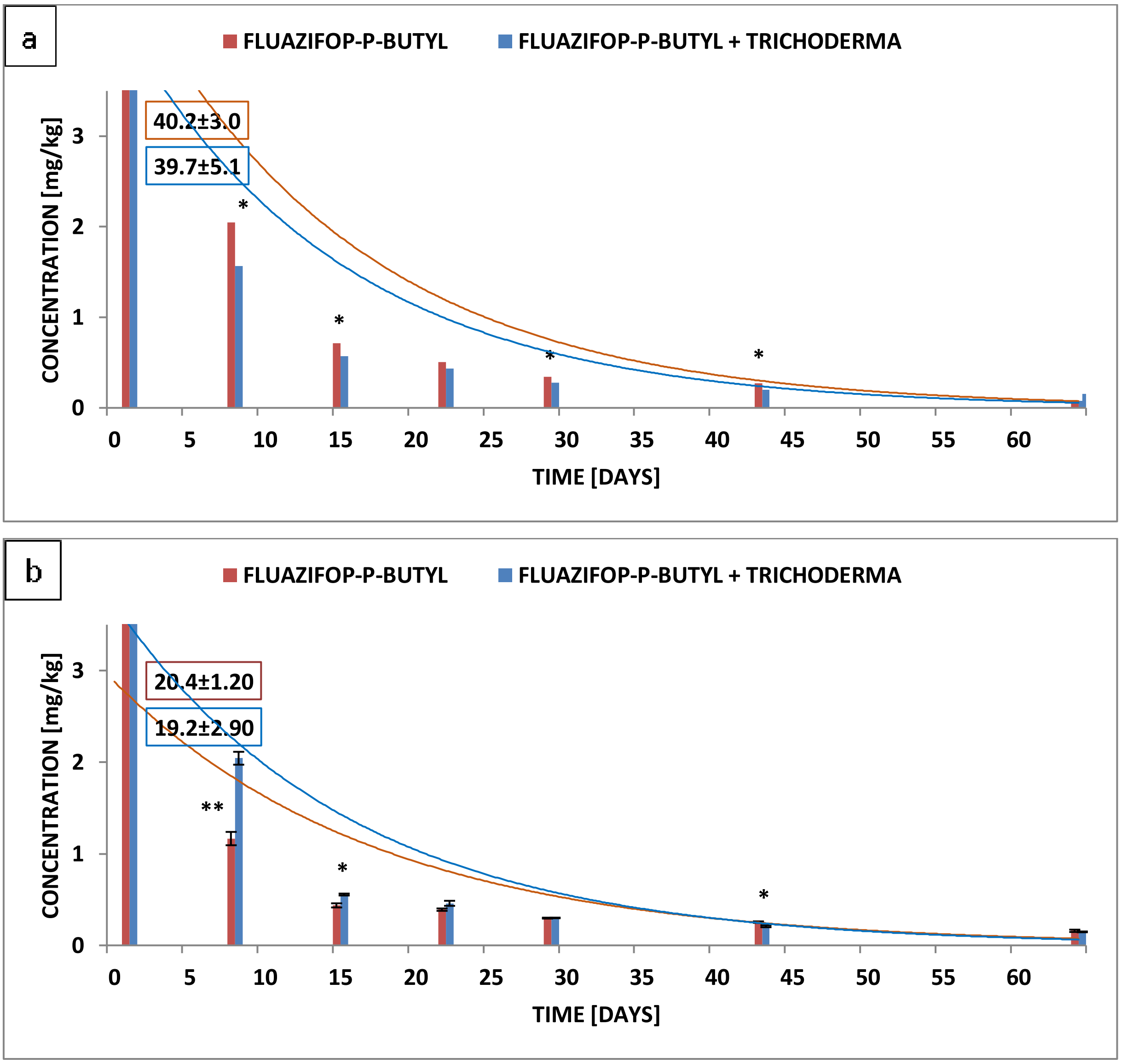

Figure S2. Plots of fluazifop-P-butyl dissipations, A - experiment 1, B - experiment 2. Statistically significant p values are shown as $p < 0.05$ (*) and $p < 0.01$ (**). Initial values of fluazifop-P-butyl are given on plots.

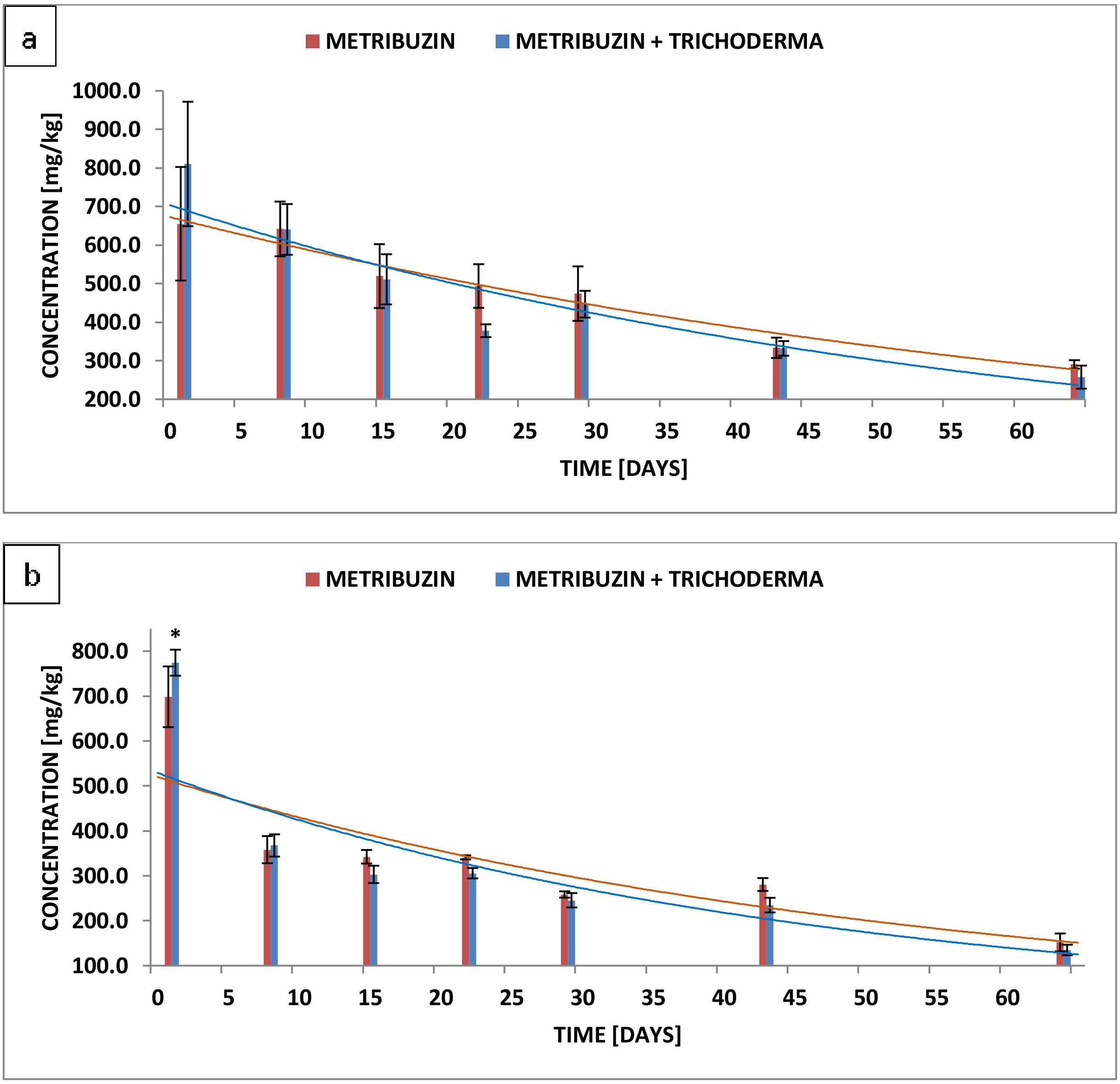

Figure S3. Plots of metribuzin dissipations, A - experiment 1, B - experiment 2. Statistically significant p value is shown as $p < 0.05$ (*).

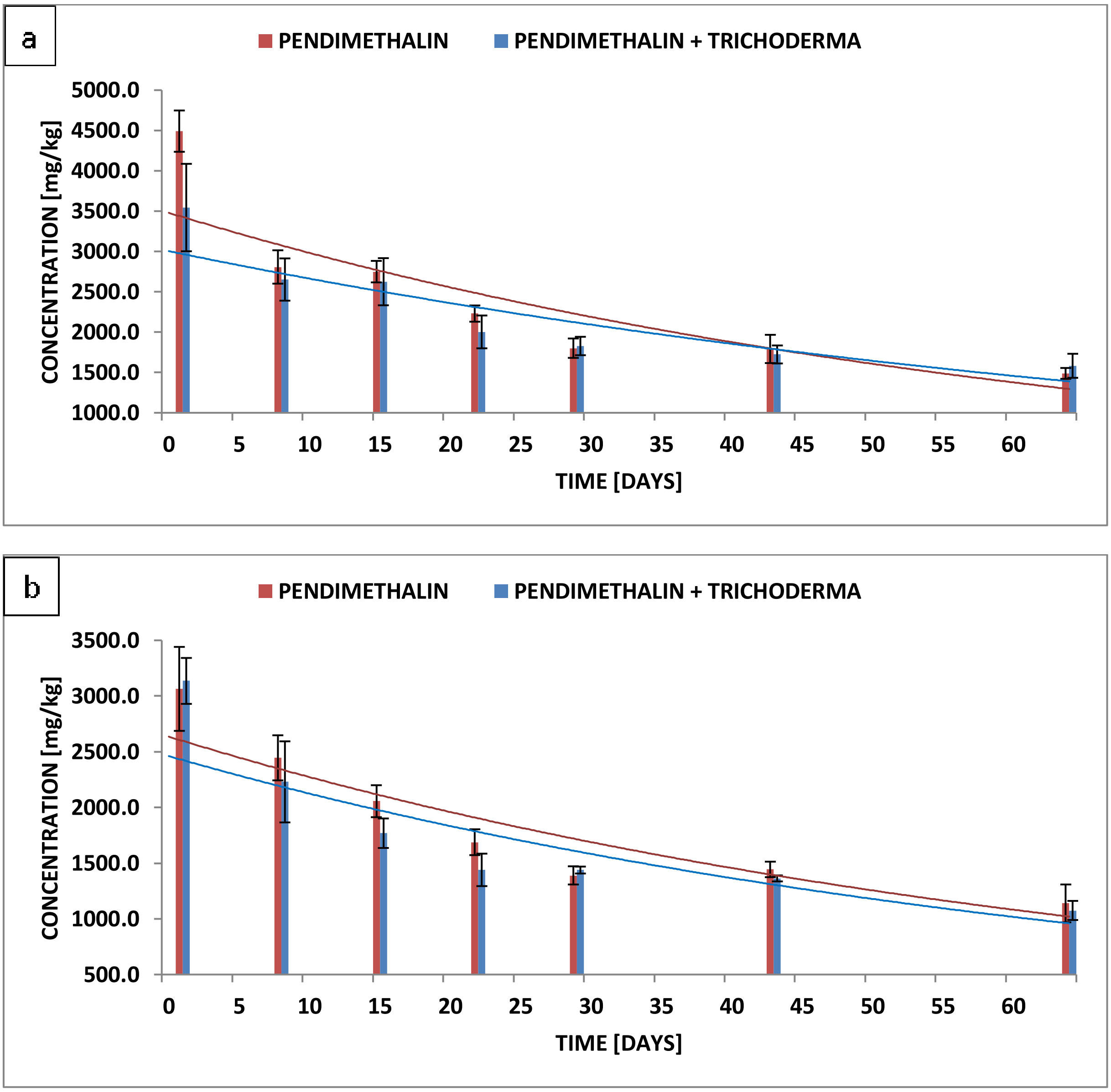

Figure S4. Plots of pendimethalin dissipations, A - experiment 1, B - experiment 2.

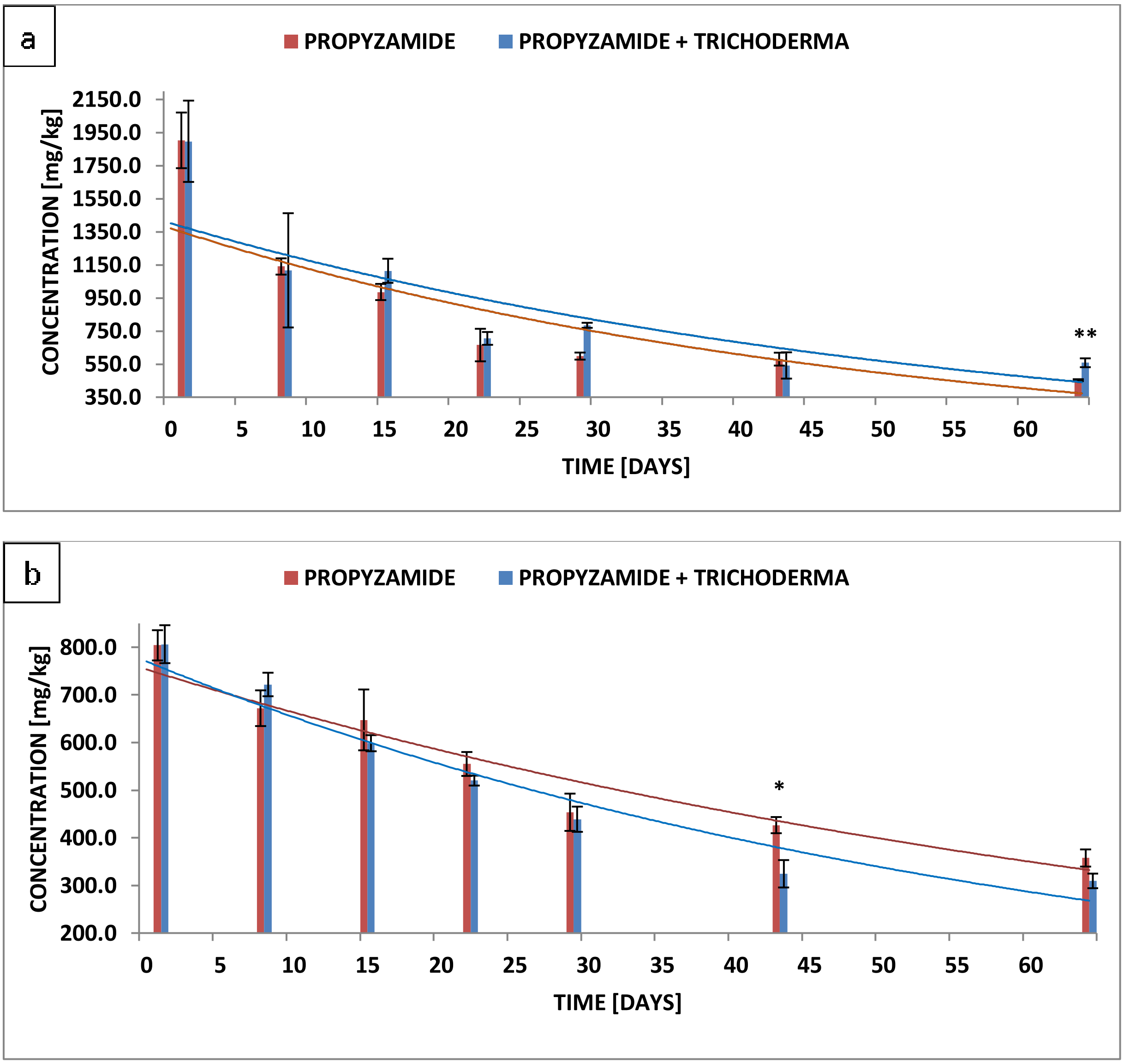

Figure S5. Plots of propyzamide dissipations, A - experiment 1, B - experiment 2. Statistically significant p values are shown as $p < 0.05$ (*) and $p < 0.01$ (**).
